# Supplementary material for: Identification and functional analysis of non-coding regulatory small RNA FenSr3 in Bacillus amyloliquefaciens LPB-18
Source: PeerJ. 2023 May 15;11:e15236. doi: 10.7717/peerj.15236 (PMC10194069; doi:10.7717/peerj.15236)
Supplement: Supplemental Information 4 [file peerj-11-15236-s004.zip › KO/CK-vs-T1_map/map05164.html]

KEGG PATHWAY: Influenza A - Reference pathway


|  |  |
| --- | --- |
| **Influenza A - Reference pathway** |  |

[
Pathway menu
| Organism menu
| Pathway entry
| Show description
| User data mapping
]

Influenza is a contagious respiratory disease caused by influenza virus infection. Influenza A virus is responsible for both annual seasonal epidemics and periodic worldwide pandemics. Novel strains that cause pandemics arise from avian influenza virus by genetic reassortment among influenza viruses and two surface glycoproteins HA and NA form the basis of serologically distinct virus types. The innate immune system recognizes invaded virus through multiple mechanisms. Viral non-structural NS1 protein is a multifunctional virulence factor that interfere IFN-mediated antiviral response. It inhibits IFN production by blocking activation of transcription factors such as NF-kappa B, IRF3 and AP1. NS1 further inhibits the activation of IFN-induced antiviral genes. PB1-F2 protein is another virulence factor that induce apoptosis of infected cells, which results in life-threatening bronchiolitis.

|  |  |  |
| --- | --- | --- |
| Reference pathway | 184% 150% 122% 100% 82% 67% 55% | 图片下载 |
